# Effects of Early Life Paracetamol Use on the Incidence of Allergic Disease and Sensitization: 5 Year Follow-Up of an Ethiopian Birth Cohort

**DOI:** 10.1371/journal.pone.0093869

**Published:** 2014-04-09

**Authors:** Alemayehu Amberbir, Girmay Medhin, Charlotte Hanlon, John Britton, Gail Davey, Andrea Venn

**Affiliations:** 1 Department of Infectious Disease Epidemiology, London School of Hygiene and Tropical Medicine, London, United Kingdom; 2 Division of Epidemiology and Public Health, University of Nottingham, Nottingham, United Kingdom; 3 Aklilu Lemma Institute of Pathobiology, Addis Ababa University, Addis Ababa, Ethiopia; 4 Department of Psychiatry, Addis Ababa University, Addis Ababa, Ethiopia; 5 Brighton & Sussex Medical School, University of Brighton, Brighton, United Kingdom; Institute for Virus Research, Laboratory of Infection and Prevention, Japan

## Abstract

**Introduction:**

The hypothesis that paracetamol, one of the most widely used medicines, may increase the risk of asthma and allergic disease is of obvious importance but prospective cohort data looking at dose and timing of exposure are lacking.

**Objective:**

The aim of the study is to investigate the role of paracetamol use in early life on the prevalence and incidence of wheeze, eczema, rhinitis and allergic sensitization, prospectively over 5 years in an Ethiopian birth cohort.

**Methods:**

In 2005/6 a birth cohort of 1006 newborns was established in Butajira, Ethiopia. Questionnaire data on allergic disease symptoms, paracetamol use and numerous potential confounders were collected at ages 1, 3 and 5, and allergen skin sensitivity measured at ages 3 and 5. Multivariate logistic regression was used to determine independent effects of paracetamol exposure on the incidence of each outcome between ages 3 and 5, and prevalence at age 5.

**Findings:**

Paracetamol use in the first 3 years of life was reported in 60% of children and was associated with increased incidence of wheeze, eczema, rhinitis and allergic sensitisation between ages 3 and 5 which was statistically significant for wheeze and eczema. High exposure (reported use in the past month at age 1 and 3) was associated with a more than 3-fold increased risk of new onset wheeze (adjusted odds ratio (OR) 3.64; 95% confidence interval, 1.34 to 9.90) compared to never users. Use in the past year at age 3 but not age 1 was associated with ORs at least as large as those for use in first year of life only. Significant positive dose-response effects of early life use were seen in relation to the prevalence of all outcomes at age 5.

**Conclusions:**

Use of paracetamol in early life is a strong risk factor for incident allergic disease in childhood.

## Introduction

A higher prevalence of asthma and allergic diseases in developed than developing countries, and in urban than rural communities, is widely reported[1]–[3]. Whilst genetics are likely to play some part in explaining these differences[4], most of the differences are thought to be due to environmental exposures[5]. One hypothesis which has gained much recent support from epidemiological studies in both developed and developing countries, is that paracetamol (acetaminophen) intake increases the risk of asthma and allergic diseases[6]–[13]. with meta-analyses of the available observational studies estimating a 63% increased risk of asthma in relation to personal paracetamol use[6], and a 21–28% increased risk of wheezing in relation to *in utero* exposure[6], [7]. However the studies to date are primarily based on cross-sectional data and conducted in developed countries, and therefore alternative explanations such as aspirin avoidance, reverse causation, recall bias, and confounding, particularly by early respiratory infections, are difficult to exclude. Of the few longitudinal studies that have been conducted, only three have looked at personal use rather than *in utero* exposure, one in adults[14] and two in children [9], [15], Lowe *et al* reported an adverse effect of paracetamol use on incident childhood asthma, but the effect diminished following control for confounding by early respiratory infections[15]. The other longitudinal study in children was our follow-up of an Ethiopian cohort, in which we also saw an adverse effect of paracetamol use in the first year of life on incident wheeze at age 3 which we concluded was unlikely to be due to reverse causation or confounding by indication; however no adverse effect on eczema was seen and other allergic outcomes were not available for analysis[9].

We now report findings from five year follow-up of this cohort that shed light on the relative importance of exposures at different time points and in different doses. The primary aims were to determine the association between early life paracetamol use (dose and timing) and incidence of wheeze, eczema, rhinitis and skin sensitisation between ages 3 and 5, as well as the prevalence of these outcomes at age 5.

## Methods

### Study setting and design

The study was a population-based prospective birth cohort of mother-child pairs followed from birth to the age of five years in Butajira, Ethiopia. The study area is predominantly rural, with a subsistence lifestyle and little motorized transport. The cohort was established in 2005 and nested in the Demographic Surveillance Site (DSS) at the Butajira Rural Health Program[16]. Details of the birth cohort, including recruitment and data collection procedures up to age 3, have been described in detail elsewhere[9], [17], [18]. In brief, all women aged 15–49 in their third trimester of pregnancy between July 2005 and February 2006 who lived in the DSS area were identified and 1065 (86% of eligible) were recruited. These women subsequently gave birth to 1006 singleton live babies who have been visited at regular intervals (birth, 2 months, and at or near their 1^st^, 3^rd^ and 5^th^ birthdays).

### Data collection

At age 5, the children were revisited and an interview-led questionnaire administered to the mother (or other guardian) in the local language (Amharic) by the fieldworker as at previous visits. The allergic disease questions, which asked about symptoms of wheeze, eczema and rhinitis ever and in the past year, were based on the International Study of Asthma and Allergies in Children (ISAAC) questionnaire[19], and were the same as those asked at previous follow-ups[9], [18]. These include: *‘Has your child ever had wheezing or whistling in their chest?*’ ‘*Has your child ever had asthma*?’, *‘Has your child ever had problems with sneezing or running nose (when not affected by cold or flu), or problems with itchy watery eyes?’* and *‘Has your child ever had an itchy skin rash which has affected the skin creases, eg, front of the elbow, behind the knees, the front of the ankles, around the neck, or around the eyes?’* At year 3 and 5 follow-ups, additional questions asking about symptoms in the past year were asked, and at age 5, symptoms in the past 2 years (since previous follow-up). Questions on paracetamol use were also identical to those asked at age 1 and 3, where the mothers were asked whether the child had taken or been given paracetamol in the past 12 months, and if so, were asked to quantify the dose of paracetamol consumed in the past month. At the 5 year follow-up, mothers were additionally asked to distinguish paracetamol from aspirin and about indications for use of paracetamol. The same range of potential confounders were collected at age 5 as at age 1 and 3, namely birth order and household size, immunizations, breastfeeding, maternal and paternal history of allergy, child's bed and mattress materials, housing construction materials, household water supply, soap use, method of waste disposal, antibiotic use, indoor fuel use, presence of insecticides and animals in home. To investigate confounding by indication, information on respiratory infection symptoms (cough, fast breathing and fever) were collected, as at year 1 and 3 (see Table S1, Table S2 and Table S3). In addition to the questionnaire, allergen skin tests to *D. pteronyssinus* and cockroach allergen (*Blatella germenica*) (Biodiagnostics, Upton-upon-Severn, UK) were carried out at age 5 using identical methods to those used at age 3[20]. Stool samples were taken from the children for geohelminth, *Helicobacter pylori* and selected intestinal microflora analysis.

### Statistical analysis

**Figure 1:**
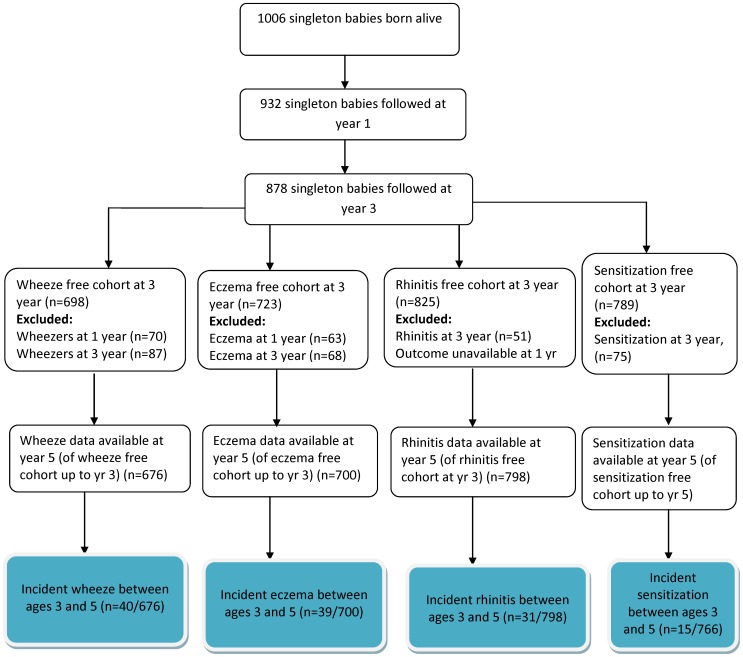
Flow chart showing reporting of symptom outcome measures and sensitization between 1 and 5 years.

The data were double-entered into EpiData version 3, cleaned, coded, and merged with the previous datasets using Stata 11 (Statacorp, College Station, TX). For longitudinal analysis, the primary outcome measures were incidence of new-onset wheeze, eczema, rhinitis and positive allergen sensitization between age 3 and 5. To compute the incidence of wheeze, a wheeze-free cohort comprising all children with a negative response to the wheeze questions at age 1 and 3 was selected, and children who reported wheeze at the 5 year follow-up were defined as having new onset wheeze. Incidence of eczema and rhinitis were computed in a similar way, although the rhinitis-free cohort at age 3 was based on responses given at age 3 only as the rhinitis questions were not asked at year 1. A positive skin test was defined as a corrected (allergen minus saline control) mean skin wheal diameter of 3 mm or greater, consistent with our previous work in the same birth cohort[20]. Those children who were negative to both *D. pteronyssinus* and cockroach allergens at age 3 were selected for analysis and new-onset sensitization defined as a positive result to either allergen at age 5 (see Figure 1). A variable called ‘early life paracetamol use’ representing paracetamol use prior to the disease outcomes was computed, with children categorised as either ‘never exposed’ (negative response at age 1 and 3), ‘exposed at year 1 not at year 3’, ‘exposed at year 3 not at year 1’, or ‘persistently exposed’ (positive response at age 1 and 3). A second variable ‘early life paracetamol dose’ was computed as a marker of dose during this time period, with children categorised as ‘low exposure’ (never reported use in the past month), ‘medium exposure’ (use in past month at age 1 or 3), or ‘high exposure’ (use in past month at both time points).

For cross-sectional analysis, the outcomes were wheeze, eczema and rhinitis in the past 12 months as reported at age 5, and sensitization to any allergens at age 5. A marker of lifetime paracetamol exposure (‘lifetime paracetamol use’) was computed with the categories ‘never exposed’ (no use in past year reported at age 1, 3 or 5), ‘past exposure but not current’ (use in past year reported at age 1 or 3 but not 5), ‘current exposure with or without past exposure’ (use in past year reported at age 5), and ‘persistent exposure’ (use in past year reported at age 1, 3 and 5). A variable reflecting total lifetime dose was also created by summing responses at each exposure time (year 1, 3 and 5), and categorising as ‘never exposed’, ‘light exposure’, ‘medium exposure’, and ‘high exposure.’

For both longitudinal and cross-sectional analyses, multivariate logistic regression was used to compute adjusted odds ratios (OR) and 95% confidence intervals (CI) for each outcome in relation to the paracetamol variables, controlling for *a priori* confounders (area of residence, child's gender, and maternal education). The effect of further control for the other variables collected, including symptoms of respiratory tract infection in the first year of life (see Table S1, Table S2 and Table S3), and other demographic and lifestyle variables collected during pregnancy and birth (birth weight, initiation of breastfeeding, and pre-lacteal feeding) were also explored. These covariates were retained in the model if they altered the odds ratios for the main exposure of interest by more than 10%. Those resulting in a 10% change or above in the size of the exposure effect were retained in the final models. The significance of the association between exposure and outcome in the model was assessed using a likelihood ratio test for the association (LRT) and 95% confidence interval with the corresponding p-value. The significance of the association between exposure and outcome in the model was assessed using the P-value from LRT. Multicollinearity among the explanatory variables was assessed and the final model fitted in the absence of any response variable that correlated to any other.

### Ethics statement

Ethical approval was granted by the National Ethical Review Committee of the Ethiopian Science and Technology Ministry and the University of Nottingham, UK. Written, informed consent was obtained from the mothers at each visit, or from the next of kin, caretakers, or guardians on behalf of the children enrolled in the study. As the majority of women or caretakers were non-literate, the information sheet and consent form was read to them and they were required to give a finger-print to signify willingness to participate. The ethics committees in Addis Ababa, Ethiopia and Nottingham, UK approved this consent procedure.

## Results

### Description of the cohort

At year five, 863 children were followed-up, representing 86% of the original cohort (n = 1006) and 98% of those followed-up at age 3 (n = 878) (Fig 1). Of these 863 children, 852 had reported outcome data. Children who were lost to follow up (less than 2% between ages 3 and 5) were not different to those followed in terms of paracetamol exposure or the outcomes collected.

### New-onset symptoms and sensitization between ages 3 and 5

Figure 1 shows that new onset wheeze between age 3 and 5 was reported in 5.9% (40/676) of the children with no reported wheeze up to age 3. Among the eczema-free cohort, incident eczema was reported in 5.8% (39/700); in the rhinitis-free cohort, incident rhinitis was reported in 3.9% (31/798); and in children not sensitized at age 3, new sensitization to either *D*. *pteronyssinus* or cockroach allergen at age 5 was found in 2.0% (15/766) (Figure 1). These incidence risks did not differ by place of residence. Sensitization at age 3 significantly increased the risk of incident eczema (OR = 2.49, 95% CI 1.04 to 5.95) and rhinitis (OR = 2.54, 95% CI 1.00 to 6.45) between ages 3 and 5, but not wheeze (OR = 1.45, 95% CI 0.54 to 3.84) (data not shown).

### Relation between potential confounders and allergic symptoms and sensitization

Infant symptoms of respiratory infections (cough, fever, and fast breathing) were significantly positively associated with incident wheeze between ages 3 and 5, but not eczema, whilst fever was positively associated with incident rhinitis and negatively with new onset sensitization (Table S2 and S3). Associations between other potential confounders and outcomes are shown in the (Table S2 and S3).

### Paracetamol use

Paracetamol use was commonly reported in the cohort with almost 60% of the children receiving paracetamol in the first three years of life, and over 35% reporting current exposure at age 5 (Table 1, 2, 3, 4). Paracetamol was the analgesic and antipyretic drug of choice among 84% of the birth cohort mothers, and most said it was readily available (77%) and affordable (92%). The majority (78%) of mothers were able to differentiate paracetamol from aspirin. Aspirin avoidance was reported by only 1.4% of the mothers because of asthma, and 1.5% due to other allergic diseases (data not shown). The most common indications for use of paracetamol were fever (31%) and headache (23.6%), with wheeze, cough and allergy indications uncommon (<4% reported each) (see Figure S1).

**Table 1:** Univariate and multivariate analysis of wheeze in relation to early and lifetime use of paracetamol up to the age of 5.

Longitudinal analysis (N = 676)							
	Wheeze never up to age 3 (N = 676)						
Exposure at age 1 and 3 yr	Overall N (%)	n (%) new wheeze	Crude OR (95% CI)	Adjusted OR* (95% CI)	P-value	Further adjusted OR† (95% CI)	P-value
Early life paracetamol use£					0.18‡		0.26‡
Never exposed	287 (42.5)	13 (4.5)	1	1		1	
Exposed at yr 1, but not at yr3	120 (17.8)	4 (3.3)	0.73 (0.23,2.78)	0.73 (0.23,2.31)		0.62 (0.20,1.96)	
Exposed at yr 3, but not at yr 1	155 (22.9)	12 (7.7)	1.77 (0.79,3.98)	1.63 (0.72,3.70)		1.49 (0.65,3.39)	
Persistently exposed	114 (16.9)	11 (9.7)	2.25 (0.98,5.18)	2.09 (0.90,4.86)		1.72 (0.74,4.03)	
Early life paracetamol dose∑					0.04‡		0.05‡
Low exposure	436 (64.5)	23 (5.3)	1	1	0.05¶	1	0.11¶
Medium exposure	208 (30.8)	11 (5.3)	1.00 (0.48,2.10)	1.04 (0.50,2.19)		0.92 (0.44,1.95)	
High exposure	32 (4.7)	6 (18.8)	4.14 (1.56,11.06)	4.08 (1.51,11.07)		3.64 (1.34,9.90)	

Cross-sectional analysis (N = 852)							
Exposure at age 1,3 and yr 5	Overall N (%)	Wheeze N (%)	Crude OR (95% CI)	Adjusted OR* (95% CI)	P-value	Further adjusted OR† (95% CI)	P-value
Lifetime paracetamol useβ					<0.01‡		0.01‡
Never exposed	258 (30.4)	4 (1.6)	1	1		1	
Past exposure but not current	285 (33.6)	10 (3.5)	2.31 (0.72,7.45)	2.38 (0.73,7.75)		2.14 (0.65,7.04)	
Current exposure ± past exposure	207 (24.4)	10 (4.8)	3.22 (1.00,10.43)	3.14 (0.97,10.20)		1.81 (0.54,6.05)	
Persistently exposed	98 (11.6)	13 (13.3)	9.71 (3.08,30.59)	10.29 (3.19,33.19)		5.87 (1.76,19.62)	
Lifetime paracetamol dose†					<0.01‡		0.01‡
Never exposed	258 (30.4)	4 (1.6)	1	1	<0.01¥	1	<0.01¥
Light exposure	286 (33.7)	8 (2.8)	1.83 (0.54,6.14)	1.83 (0.54,6.20)		1.50 (0.44,5.13)	
Medium exposure	190 (22.4)	11 (5.8)	3.90 (1.22,12.45)	3.90 (1.22,12.47)		2.55 (0.78,8.35)	
High exposure	114 (13.4)	14 (12.3)	8.89 (2.86,27.66)	8.96 (2.85,28.23)		5.03 (1.54,16.37)	

*ORs adjusted for child's gender, area of residence and maternal education.

† ORs adjusted for child's gender, area of residence and maternal education and additionally adjusted for symptoms of respiratory infections in the first year of life (and for cross-sectional analysis reported infection at year 5).

‡ Overall p-value (likelihood ratio test).

¶ P value for trend computed for dose of paracetamol use in the past month: low exposure (never reported use in the past month), medium exposure (use in past month at age 1 & 3), high exposure (use in past month at both time points).

¥ P value for trend as dose of paracetamol computed using a composite score of paracetamol exposure from yr 1, 3 and 5

£ Early life paracetamol use: refers to use prior to the disease outcomes (i.e. first three years of life).

∑ Early life paracetamol dose: refers to dose of exposure in past month in first three years of life prior to disease outcomes.

β Lifetime paracetamol use: refers to use of paracetamol up to the age of 5 (i.e. ages 1, 3 and 5).

± Lifetime paracetamol dose: refers to dose of paracetamol exposure up to the age of 5 (i.e. ages 1, 3 and 5).

**Table 2:** Univariate and multivariate analysis of eczema in relation to early and lifetime use of paracetamol up to the age of 5.

Longitudinal analysis (N = 700)							
	Eczema never up to age 3 (N = 700)						
Exposure at age 1 and 3 yr	Overall N (%)	n (%) new eczema	Crude OR (95% CI)	Adjusted OR* (95% CI)	P-value	Further adjusted OR† (95% CI)	P-value
Early life paracetamol use£					0.01‡		0.02‡
Never exposed	275 (39.3)	6 (2.2)	1	1		1	
Exposed at yr 1, but not at yr3	123 (17.6)	8 (6.5)	3.12 (1.06,9.19)	3.11 (1.05,9.18)		3.01 (1.00,9.04)	
Exposed at yr 3, but not at yr 1	161 (23.0)	13 (8.1)	3.94 (1.47,10.58)	3.75 (1.39,10.12)		3.70 (1.37,10.01)	
Persistently exposed	141 (20.1)	12 (8.5)	4.17 (1.53,11.36)	3.96 (1.44,10.89)		3.82 (1.36,10.73)	
Early life paracetamol dose∑					0.04‡		0.08‡
Low exposure	423 (60.4)	16 (3.8)	1	1	0.05¶	1	0.06¶
Medium exposure	230 (32.9)	20 (8.7)	2.42 (1.23,4.77)	2.39 (1.21,4.71)		2.31 (1.16,4.60)	
High exposure	47 (6.7)	3 (6.4)	1.73 (0.49,6.19)	1.65 (0.46,5.93)		1.59 (0.44,5.74)	

Cross-sectional analysis (N = 852)							
Exposure at age 1,3 and yr 5	Overall N (%)	Eczema n (%)	Crude OR (95% CI)	Adjusted OR* (95% CI)	P-value	Further adjusted OR† (95% CI)	P-value
Lifetime paracetamol useβ					<0.01‡		<0.01‡
Never exposed	258 (30.4)	4 (1.6)	1	1		1	
Past exposure but not current	285 (33.6)	7 (2.5)	1.60 (0.46,5.53)	1.66 (0.48,5.76)		1.61 (0.46,5.61)	
Current exposure ± past exposure	207 (24.4)	17 (8.2)	5.68 (1.88,17.16)	6.00 (1.98,18.19)		5.29 (1.67,16.80)	
Persistently exposed	98 (11.6)	8 (8.2)	5.64 (1.66,19.20)	6.12 (1.77,21.13)		5.40 (1.50,19.45)	
Lifetime paracetamol dose†					<0.01‡		0.01‡
Never exposed	258 (30.4)	4 (1.6)	1	1	<0.01¥	1	<0.01¥
Light exposure	286 (33.7)	11 (3.9)	2.54 (0.80,8.08)	2.71 (0.85,8.66)		2.53 (0.79,8.13)	
Medium exposure	190 (22.4)	9 (4.7)	3.16 (0.96,10.41)	3.26 (0.98,10.77)		2.78 (0.82,9.45)	
High exposure	114 (13.4)	12 (10.5)	7.47 (2.35,23.70)	8.27 (2.57,26.58)		6.70 (1.99,22.54)	

*ORs adjusted for child's gender, area of residence and maternal education.

† ORs adjusted for child's gender, area of residence and maternal education and additionally adjusted for symptoms of respiratory infections in the first year of life (and for cross-sectional analysis reported infection at year 5).

‡ Overall p-value (likelihood ratio test).

¶ P value for trend computed for dose of paracetamol use in the past month: low exposure (never reported use in the past month), medium exposure (use in past month at age 1 & 3), high exposure (use in past month at both time points).

¥ P value for trend as dose of paracetamol computed using a composite score of paracetamol exposure from yr 1, 3 and 5.

£ Early life paracetamol use: refers to use prior to the disease outcomes (i.e. first three years of life).

∑ Early life paracetamol dose: refers to dose of exposure in past month in first three years of life prior to disease outcomes.

β Lifetime paracetamol use: refers to use of paracetamol up to the age of 5 (i.e. ages 1, 3 and 5).

± Lifetime paracetamol dose: refers to dose of paracetamol exposure up to the age of 5 (i.e. ages 1, 3 and 5).

**Table 3:** Univariate and multivariate analysis of rhinitis in relation to early and lifetime use of paracetamol up to the age of 5.

Longitudinal analysis (N = 798)							
	Rhinitis never up to age 3 (N = 798)						
Exposure at age 1 and 3 yr	Overall N (%)	n (%) new rhinitis	Crude OR (95% CI)	Adjusted OR* (95% CI)	P-value	Further adjusted OR† (95% CI)	P-value
Early life paracetamol use£					0.04‡		0.07‡
Never exposed	319 (40.0)	5 (1.6)	1	1		1	
Exposed at yr 1, but not at yr3	138 (17.3)	6 (4.4)	2.85 (0.86,9.52)	2.75 (0.82,9.20)		2.42 (0.72,8.14)	
Exposed at yr 3, but not at yr 1	182 (22.8)	11 (6.0)	4.04 (1.38,11.82)	3.96 (1.35,11.65)		3.74 (1.27,11.04)	
Persistently exposed	159 (19.9)	9 (5.7)	3.77 (1.24,11.44)	3.60 (1.18,11.03)		3.10 (1.00,9.57)	
Early life paracetamol dose∑					0.10‡		0.18‡
Low exposure	485 (60.8)	13 (2.7)	1	1	0.04¶	1	0.07¶
Medium exposure	258 (32.3)	14 (5.4)	2.08 (0.96,4.50)	2.07 (0.95,4.47)		1.90 (0.87,4.12)	
High exposure	55 (6.9)	4 (7.3)	2.85 (0.90,9.06)	2.61 (0.82,8.39)		2.31 (0.72,7.46)	

Cross-sectional analysis (N = 852)							
Exposure at age 1,3 and yr 5	Overall N (%)	Rhinitis n (%)	Crude OR (95% CI)	Adjusted OR* (95% CI)	P-value	Further adjusted OR† (95% CI)	P-value
Lifetime paracetamol useβ					<0.01‡		0.07‡
Never exposed	258 (30.4)	2 (0.9)	1	1		1	
Past exposure but not current	285 (33.6)	9 (3.2)	4.17 (0.89,19.50)	4.10 (0.87,19.21)		3.60 (0.76,17.01)	
Current exposure ± past exposure	207 (24.4)	11 (5.3)	7.18 (1.57, 32.78)	7.09 (1.55,32.38)		3.82 (0.82,17.82)	
Persistently exposed	98 (11.6)	9 (9.2)	12.94 (2.74,61.05)	12.58 (2.63,60.02)		6.59 (1.34,32.35)	
Lifetime paracetamol dose†					<0.01‡		0.01‡
Never exposed	258 (30.4)	2 (0.9)	1	1	<0.01¥	1	<0.01¥
Light exposure	286 (33.7)	7 (2.5)	3.21 (0.66,15.60)	3.16 (0.65,15.38)		2.48 (0.50,12.25)	
Medium exposure	190 (22.4)	10 (5.3)	7.11 (1.54,32.84)	7.01 (1.52,32.44)		4.36 (0.93,20.55)	
High exposure	114 (13.4)	12 (10.5)	15.06 (3.31,68.47)	14.66 (3.20,67.10)		7.65 (1.63,35.97)	

*ORs adjusted for child's gender, area of residence and maternal education.

† ORs adjusted for child's gender, area of residence and maternal education and additionally adjusted for symptoms of respiratory infections in the first year of life (and for cross-sectional analysis reported infection at year 5).

‡ Overall p-value (likelihood ratio test).

¶ P value for trend computed for dose of paracetamol use in the past month: low exposure (never reported use in the past month), medium exposure (use in past month at age 1 & 3), high exposure (use in past month at both time points).

¥ P value for trend as dose of paracetamol computed using a composite score of paracetamol exposure from yr 1, 3 and 5.

£ Early life paracetamol use: refers to use prior to the disease outcomes (i.e. first three years of life).

∑ Early life paracetamol dose: refers to dose of exposure in past month in first three years of life prior to disease outcomes.

β Lifetime paracetamol use: refers to use of paracetamol up to the age of 5 (i.e. ages 1, 3 and 5).

± Lifetime paracetamol dose: refers to dose of paracetamol exposure up to the age of 5 (i.e. ages 1, 3 and 5).

**Table 4:** Univariate and multivariate analysis of sensitization in relation to early and lifetime use of paracetamol up to the age of 5.

Longitudinal analysis (N = 766)							
	Sensitization never up to age 3 (N = 766)						
Exposure at age 1 and 3 yr	Overall N (%)	n (%) new atopy	Crude OR (95% CI)	Adjusted OR* (95% CI)	P-value	Further adjusted OR† (95% CI)	P-value
Early life paracetamol use£					0.78‡		0.59‡
Never exposed	290 (38.0)	5 (1.7)	1	1		1	
Exposed at yr 1, but not at yr3	133 (17.4)	2 (1.5)	0.87 (0.17,4.54)	0.85 (0.16,4.42)		1.13 (0.21,6.23)	
Exposed at yr 3, but not at yr 1	176 (23.0)	3 (1.7)	0.99 (0.23,4.19)	0.98 (0.23,4.17)		1.09 (0.25,4.70)	
Persistently exposed	165 (21.6)	5 (3.0)	1.78 (0.51,6.25)	1.73 (0.49,6.11)		2.48 (0.63,9.76)	
Early life paracetamol dose∑					0.72‡		0.57‡
Low exposure	446 (58.4)	8 (1.8)	1	1	0.51¶	1	0.31¶
Medium exposure	264 (34.6)	5 (1.9)	1.06 (0.34,3.26)	1.05 (0.34,3.26)		1.26 (0.40,4.04)	
High exposure	54 (7.1)	2 (3.7)	2.11 (0.44,10.18)	1.99 (0.41,9.70)		2.59 (0.50,13.29)	

Cross-sectional analysis (N = 841)							
Exposure at age 1,3 and yr 5	Overall N (%)	Atopy n (%)	Crude OR (95% CI)	Adjusted OR* (95% CI)	P-value	Further adjusted OR† (95% CI)	P-value
Lifetime paracetamol useβ					0.54‡		0.46‡
Never exposed	254 (30.2)	4 (1.6)	1	1		1	
Past exposure but not current	283 (33.7)	5 (1.8)	1.12 (0.30,4.23)	1.15 (0.30,4.39)		1.19 (0.31,4.53)	
Current exposure ± past exposure	206 (24.5)	4 (1.9)	1.24 (0.31, 5.01)	1.26 (0.31,5.12)		1.48 (0.34,6.44)	
Persistently exposed	98 (11.7)	4 (4.1)	2.66 (0.65,10.85)	2.83 (0.68,11.86)		3.35 (0.74,15.11)	
Lifetime paracetamol dose†					0.13‡		0.09‡
Never exposed	254 (30.2)	4 (1.6)	1	1	0.06¥	1	0.04¥
Light exposure	285 (33.9)	4 (1.4)	0.89 (0.22,3.59)	0.93 (0.23,3.78)		0.99 (0.24,4.06)	
Medium exposure	189 (22.5)	3 (1.6)	1.01 (0.22,4.56)	1.01 (0.22,4.58)		1.20 (0.25,5.64)	
High exposure	113 (13.4)	6 (5.3)	3.50 (0.97,12.67)	3.71 (1.01,13.68)		4.80 (1.17,19.68)	

*ORs adjusted for child's gender, area of residence and maternal education.

† ORs adjusted for child's gender, area of residence and maternal education and additionally adjusted for symptoms of respiratory infections in the first year of life (and for cross-sectional analysis reported infection at year 5).

‡ Overall p-value (likelihood ratio test).

¶ P value for trend computed for dose of paracetamol use in the past month: low exposure (never reported use in the past month), medium exposure (use in past month at age 1 3), high exposure (use in past month at both time points).

¥ P value for trend as dose of paracetamol computed using a composite score of paracetamol exposure from yr 1, 3 and 5.

£ Early life paracetamol use: refers to use prior to the disease outcomes (i.e. first three years of life).

∑ Early life paracetamol dose: refers to dose of exposure in past month in first three years of life prior to disease outcomes.

β Lifetime paracetamol use: refers to use of paracetamol up to the age of 5 (i.e. ages 1, 3 and 5).

± Lifetime paracetamol dose: refers to dose of paracetamol exposure up to the age of 5 (i.e. ages 1, 3 and 5).

### Longitudinal associations between paracetamol use and incidence of allergic outcomes

Having adjusted for *a priori* confounders, early life paracetamol use was significantly associated with an increased risk of incident eczema and rhinitis between ages 3 and 5 (Table 2 and 3). For incident eczema, ORs for all exposed groups were significantly increased relative to the never exposed (overall p = 0.01), the highest OR being in relation to persistent exposure (OR = 3.96; 95% CI 1.44 to 10.89), and almost as high for exposure at year three but not at year one (OR = 3.75; 95% CI 1.39, 10.12) as for exposure at year one but not year three (OR = 3.11; 95% CI 1.05, 9.18) (Table 2). Similarly, for incident rhinitis, the ORs, all compared with never users, were higher for heavily exposed children (OR = 3.60; 95% CI 1.18, 11.03) and those exposed at age 3 but not age 1 (OR = 3.96; 95% CI 1.35, 11.65) than exposure at age 1 but not age 3 (OR = 2.75; 95% CI 0.82, 9.20; overall p = 0.04; Table 3). For incident wheeze and sensitization, no overall significant effect of the early life paracetamol use variable was seen, although adjusted ORs in relation to persistent exposure (reported use at age 1 and 3) were increased (Tables 1 and 4).

In relation to early life paracetamol dose, significant positive associations were seen for new-onset wheeze, eczema and rhinitis (Tables 1, 2, 3, 4). For wheeze, the OR was increased for high dose exposure only compared to low (adjusted OR = 4.08; 95% CI 1.51, 11.07), whereas for eczema and rhinitis, ORs were increased for medium and high exposure in a more dose dependent manner (p trend = 0.05 and 0.04, respectively; Tables 1, 2, 3). Incident sensitization was increased amongst those with high compared to low exposure (OR = 1.99; 95% CI 0.41, 9.70), but not to the point of statistical significance (Table 4).

Additional control for confounding by respiratory tract infections only marginally reduced the strength of the associations which remained statistically significant (Tables 1, 2, 3, 4). Further adjustment for other potential confounders including environmental and lifestyle factors in Table S2 and Table S3 did not materially alter the associations.

### Cross-sectional associations between paracetamol and allergic outcomes at age 5

Tables 1, 2, 3, 4 show significant positive effects of ‘lifetime paracetamol use’ on the prevalence of all outcomes, except sensitization, at age 5. ORs, adjusted for *a priori* confounders, were increased for all exposure categories, but tended to be highest in relation to persistent use (adjusted ORs ranging from 6.1 to 12.6) and least elevated in the children exposed in the past but not currently (adjusted ORs ranging from 1.7 to 4.1). Analyses of the lifetime paracetamol dose variable revealed significant dose dependent associations for all symptom outcomes (p<0.01) and a borderline significant association for sensitization (p = 0.06). Further adjustment for potential confounders, including symptoms of current respiratory tract infections, did not materially alter these cross-sectional associations.

## Discussion

We have found an adverse effect of early paracetamol use on risk of incident wheeze, with the heaviest users of paracetamol in the first three years of life having a nearly four-fold increased risk of new onset wheeze between age 3 and 5 compared to never users. We have also found strong and consistent adverse effects on incident eczema, not found at our previous follow-up[9], with increased risks not just restricted to the heaviest or most persistent users. For the first time, we were able to look at incident rhinitis and sensitization, revealing associations in the expected direction and of borderline significance for rhinitis but not reaching significance for sensitization. Our longitudinal findings are supported by significant, positive effects on all outcomes in cross-sectional analyses with clearer dose-response patterns seen and the advantage of greater statistical power.

An important strength of this study is the prospective design, with exposures measured prior to the onset of the outcomes, thereby helping to exclude reverse causation as a possible explanation. The design also allowed timing of exposures to be explored, and in particular, the relative importance of exposure in the first year of life, thought to be the important window of life in terms of immune development[21]. Contrary to this hypothesis, we observed equally large adverse effects on new onset disease amongst those exposed only in the second and third year of life; this also suggests that effects of personal exposure are not simply being driven by confounding by *in utero* exposure. Selection bias is unlikely to be important since we have used a population-based unselected birth cohort and only 6% of surviving mother-child dyads were lost to follow-up between birth and age 5. The low income rural setting of the cohort also has the advantage of reducing exposure misclassification since multiple formulations of paracetamol and other non-aspirin non-steroidal anti-inflammatory drugs (NSAIDs) are uncommon. Consistent with previous research in this setting[8], we found the mothers' ability to distinguish between paracetamol and aspirin to be high (78%), and reported aspirin avoidance due to awareness of contraindications in asthma to be very low (2%). A further strength of the study was control for multiple potential confounders including confounding by indication which can arise if children with early life respiratory tract infections, who may also be more susceptible to subsequently develop asthma and allergic conditions[22], are given paracetamol for symptoms of these infections. We have found that our associations generally persisted after control for such symptoms in the first year of life, though the magnitudes of effect were slightly reduced.

A potential limitation of the study is that misclassification of symptom outcomes may have arisen through the use of a questionnaire. However, the increased risk of new onset symptom outcomes in relation to sensitization at the age of 3 consistent with our previous findings[18], implies the dominance of an allergic phenotype in the cohort. The questionnaire was administered by individuals known to the mothers, and used common local terms (for example ‘*sit sit*’ for wheezing symptoms, an onomatopoeic local term) which should increase the accuracy of reporting.

Our findings fit with a recent meta-analysis of studies reporting a 63% increased risk of asthma associated with paracetamol use in children and adults[6]. Prospective evidence comes primarily from a handful of studies showing a positive relation between paracetamol exposure *in utero*, particularly in late pregnancy, and the risk of allergic diseases in childhood [13], [23], [24]. A recent meta-analysis of the available observational studies reporting exposure *in utero* and risk of childhood wheeze demonstrated a pooled OR of 1.21 (95% CI, 1.02, 1.44), irrespective of gestational age[7], which also fits with the previous meta-analysis that reported a pooled odds ratio of 1.50[6]. The only longitudinal study of personal intake in children reported an increased risk of wheeze in relation to paracetamol use in early life but, unlike in our study, the association did not remain after adjustment for respiratory tract infections[15]. However bias associated with over-adjustment in the model is difficult to exclude and since the children studied had parents with asthma and allergy[15], the associations may be confounded by genetic susceptibility. Randomised controlled trials (RCT) are scarce although one comparing paracetamol with ibuprofen showed children who took paracetamol had higher rates of outpatient visits for asthma than those taking ibuprofen[25]. However, the trial was not placebo-controlled and a true effect is difficult to untangle[25].

Evidence from *in vivo* and *in vitro* investigations supports these epidemiological observations. Therapeutic doses of paracetamol have been shown to reduce serum total antioxidant capacity within 14 days[26], possibly through a reduction in glutathione (GSH) levels[26], [27], with the lung bearing the brunt of this depletion, which predisposes it to toxic agents[28]. Glutathione depletion may also cause a shift from Th_1_ to Th_2_ cytokine production favouring allergic disorders[29]. Paracetamol may also contribute to the increased risk of asthma and COPD, via its reactive metabolite NAPQI through TRPA1-dependent neurogenic inflammatory responses in the airways[30].

In conclusion, we have found that early life exposure to paracetamol increases the risks of wheeze and allergic disease in a developing country birth cohort in which alternative explanations, including reverse causation and confounding by indication, are unlikely to play a role. As most of the evidence to date comes from observational studies, an RCT is needed to generate definitive evidence before reviewing use in children. The questions that need to be addressed in RCTs are whether paracetamol causes asthma, and whether paracetamol exacerbates asthma. Designs for these might present ethical dilemmas, since the former ideally requires a controlled trial in pregnancy (or in children) and the latter a design in which the hypothesis is that active treatment makes asthma worse; the alternative might therefore be trial designs involving withdrawal of paracetamol in relevant groups using non-aspirin NSAIDs analgesics as placebo.

## Supporting Information

Figure S1 **Indications for child's use of paracetamol at the age of 5 year.**
(TIF)

Table S1 **Summary of relevant variables collected at different time points in the cohort.**
(DOC)

Table S2 **Distribution of potential confounders in the first year of life in relation to incident symptoms outcomes between ages 3 and 5.**
(DOC)

Table S3 **Distribution of potential confounders in the first year of life in relation to incident sensitization between ages 3 and 5.**
(DOC)

## References

[1] Eder W, Ege MJ, von Mutius E (2006) The Asthma Epidemic. *N Engl J Med* 355(21): 2226–2235. 17124020 10.1056/NEJMra054308

[2] Yemaneberhan H, Bekele Z, Venn A, Lewis S, Parry E, et al (1997) Prevalence of wheeze and asthma and relation to atopy in urban and rural Ethiopia. *Lancet* 350(9071): 85–90. 9228959 10.1016/S0140-6736(97)01151-3

[3] Asher MI, Montefort S, Bjorksten B, Lai CKW, Strachan DP, et al (2006) Worldwide time trends in the prevalence of symptoms of asthma, allergic rhinoconjunctivitis, and eczema in childhood: ISAAC Phases One and Three repeat multicountry cross-sectional surveys. *Lancet* 368(9537): 733–743. 16935684 10.1016/S0140-6736(06)69283-0

[4] Moffat MF, Gut IG, Demenais F, Strachan DP, Bouzigon E, et al (2010) A Large-Scale, Consortium-Based Genome wide Association Study of Asthma. *N Engl J Med* 363(13): 1211–1221. 20860503 10.1056/NEJMoa0906312 PMC4260321

[5] Ege MJ, Mayer M, Normand AC, Genuneit J, Cookson W, et al (2011) Exposure to Environmental Microorganisms and Childhood Asthma. *N Engl J Med* 364(8): 701–709. 21345099 10.1056/NEJMoa1007302

[6] Etminan M, Sadatsafavi M, Jafari S, Doyle-Waters M, FitzGerald JM (2009) Acetaminophen use and the risk of asthma in children and adults. A systematic review and meta analysis. *Chest* 136: 1316–1323. 19696122 10.1378/chest.09-0865

[7] Eyers S, Weatherall M, Jefferies S, Beasley R (2011) Paracetamol in pregnancy and the risk of wheezing in offspring: a systematic review and meta-analysis. *Clin Exp Allergy* 41(4): 482–489. 21338428 10.1111/j.1365-2222.2010.03691.x

[8] Davey G, Berhane Y, Duncan P, ref-Adib G, Britton J, et al (2005) Use of acetaminophen and the risk of self-reported allergic symptoms and skin sensitization in Butajira, Ethiopia. *J Allergy Clin Immunol* 116(4): 863–868. 16210062 10.1016/j.jaci.2005.05.045

[9] Amberbir A, Medhin G, Alem A, Britton J, Davey G, et al (2011) The role of acetaminophen and geohelminth infection on the incidence of wheeze and eczema: a longitudinal birth-cohort study. *Am J Respir Crit Care Med* 183(2): 165–170. 20935107 10.1164/rccm.201006-0989OC PMC3040388

[10] Farquhar H, Stewart A, Mitchell E, Crane J, Eyers S, et al (2010) The role of paracetamol in the pathogenesis of asthma. *Clin Exp Allergy* 40: 32–41. 20205695 10.1111/j.1365-2222.2009.03378.x

[11] Beasley RW, Clayton TO, Crane J, Crane J, Lai CKW, et al (2011) Acetaminophen use and risk of asthma, rhinoconjunctivitis, and eczema in adolescents: International Study of Asthma and Allergies in Childhood Phase Three. *Am J Respir Crit Care Med* 183(2): 171–178. 20709817 10.1164/rccm.201005-0757OC

[12] Beasley R, Clayton T, Crane J, von Mutius E, Lai CKW, et al (2008) Association between paracetamol use in infancy and childhood, and risk of asthma, rhinoconjunctivitis, and eczema in children aged 6–7 years: analysis from Phase Three of the ISAAC programme. *Lancet* 372(9643): 1039–1048. 18805332 10.1016/S0140-6736(08)61445-2

[13] Shaheen SO, Newson RB, Sherriff A, Henderson AJ, Heron JE, et al (2002) Paracetamol use in pregnancy and wheezing in early childhood. *Thorax* 57(11): 958–963. 12403878 10.1136/thorax.57.11.958 PMC1746229

[14] Barr RG, Wentowski CC, Curhan GC, Somers SC, Stampfer MJ, et al (2004) Prospective study of acetaminophen use and newly diagnosed asthma among women. *Am J Respir Crit Care Med* 169(7): 836–841. 14711794 10.1164/rccm.200304-596OC

[15] Lowe AJ, Carlin JB, Bennett CM, Hosking CS, Allen KJ, et al (2010). Paracetamol use in early life and asthma: prospective birth cohort study. *BMJ*; 341 (c4616). 10.1136/bmj.c4616 PMC2939956 20843914

[16] Berhane Y, Wall S, Kebede D, Emmelin A, Enquselassie F, et al (1999) Establishing an epidemiological field laboratory in rural areas-potential for public health research and interventions. Ethiop J Health Dev 13: 1–47.

[17] Hanlon C, Medhin G, Alem A, Tesfaye F, Lakew Z, et al (2009) Impact of antenatal common mental disorders upon perinatal outcomes in Ethiopia: the P-MaMiE population-based cohort study. *Trop Med Int Health* 14(2): 156–166. 19187514 10.1111/j.1365-3156.2008.02198.x

[18] Belyhun Y, Amberbir A, Medhin G, Erko B, Hanlon C, et al (2010) Prevalence and risk factors of wheeze and eczema in one year old children: the Butajira birth cohort, Ethiopia. *Clin Exp Allergy* (40): 619–626. 20447078 10.1111/j.1365-2222.2010.03479.x

[19] Asher MI, Keil U, Anderson HR, Beasley R, Crane J, et al (1995) International Study of Asthma and Allergies in Childhood (ISAAC): Rationale and methods. *Eur Respir J* 8: 483–491. 7789502 10.1183/09031936.95.08030483

[20] Amberbir A, Medhin G, Erku W, Alem A, Simms R, et al (2011) Effects of Helicobacter pylori, geohelminth infection and selected commensal bacteria on the risk of allergic disease and sensitization in 3-year-old Ethiopian children. Clin Exp Allergy 41(10): 1422–1430. 21831135 10.1111/j.1365-2222.2011.03831.x

[21] Prescott SL, Macaubas C, Smallacombe T, Holt BJ, Sly PD, et al (1999) Development of allergen-specific T-cell memory in atopic and normal children. *Lancet* 353(9148): 196–200. 9923875 10.1016/S0140-6736(98)05104-6

[22] Illi S, von Mutius E, Lau S, Bergmann, Niggemann B, et al (2001) Early childhood infectious diseases and the development of asthma up to school age: a birth cohort study. *BMJ* 322(7283): 390–395. 11179155 10.1136/bmj.322.7283.390 PMC26566

[23] Rebordosa C, Kogevinas M, Sorensen HT, Olsen J (2008) Pre-natal exposure to paracetamol and risk of wheezing and asthma in children: A birth cohort study. *Int J Epidemiol* 37(3): 583–590. 18400839 10.1093/ije/dyn070

[24] Perzanowski MS, Miller RL, Ali DB, Garfinkel RS, Chew GL, et al (2008) Prenatal acetaminophen use is a risk for wheeze at age 5 years in a low income urban population with a high risk for asthma. *J Allergy Clin Immunol*; (Supp 1):S231.

[25] Lesko SM, Louik C, Vezina RM, Mitchell AA (2002) Asthma morbidity after the short-term use of ibuprofen in children. *Pediatrics* 109(2): e20. 11826230 10.1542/peds.109.2.e20

[26] Nuttall SL, Khan JN, Thorpe GH, Langford N, Kendall MJ (2003) The impact of therapeutic doses of paracetamol on serum total antioxidant capacity. *J Clin Pharm Ther* 28(4): 289–294. 12911681 10.1046/j.1365-2710.2003.00493.x

[27] Nuttall SL, Williams J, Kendall MJ (2003) Does paracetamol cause asthma? *J Clin Pharm Ther* 28(4): 251–257. 12911676 10.1046/j.1365-2710.2003.00492.x

[28] Micheli L, Cerretani D, Fiaschi A, Gioorgi G, Romeo R, et al (1994) Effect of acetaminophen on glutathione levels in rat testis and lung. *Environ Health Perspect*; (Supp 9):63–64. 10.1289/ehp.94102s963 PMC1566779 7698087

[29] Peterson JD, Herzenberg LA, Vasquez K, Waltenbaugh C (1998) Glutathione levels in antigen-presenting cells modulate Th1 versus Th2 response patterns. Proceedings of the National Academy of Sciences of the United States of America 95(6): 3071–3076. 9501217 10.1073/pnas.95.6.3071 PMC19696

[30] Nassini R, Materazzi S, Andre E, Sartiani L, Aldini G, et al (2010) Acetaminophen, via its reactive metabolite N-acetyl-p-benzo-quinoneimine and transient receptor potential ankyrin-1 stimulation, causes neurogenic inflammation in the airways and other tissues in rodents. *FASEB J* 24(12): 4904–4916. 20720158 10.1096/fj.10-162438

