# Supplementary figures and images for: Effects of Early Life Paracetamol Use on the Incidence of Allergic Disease and Sensitization: 5 Year Follow-Up of an Ethiopian Birth Cohort
Source: PLoS One. 2014 Apr 9;9(4):e93869. doi: 10.1371/journal.pone.0093869 (PMC3981735; doi:10.1371/journal.pone.0093869)

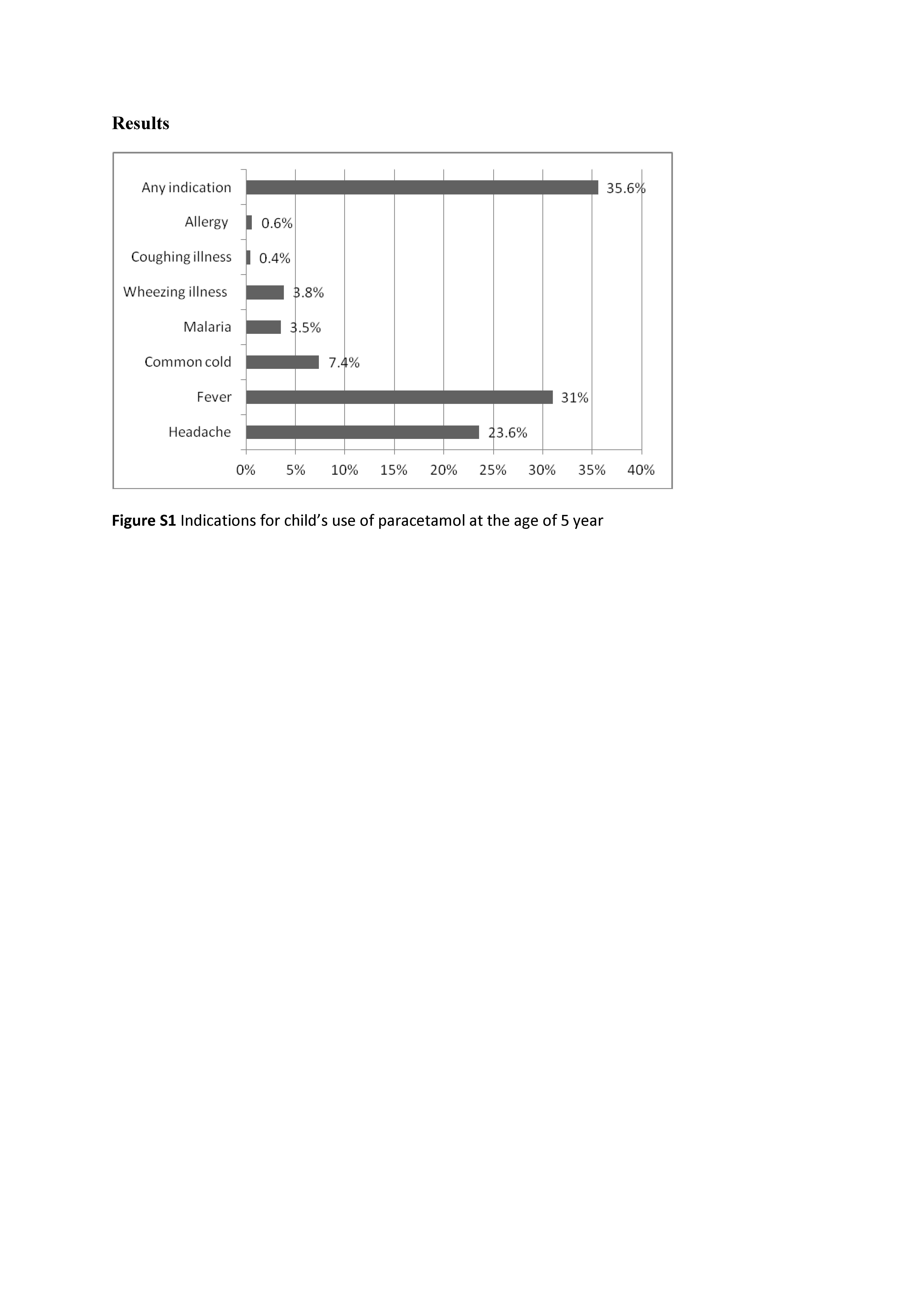

Supplement: Figure S1 — Indications for child's use of paracetamol at the age of 5 year. (TIF) [file pone.0093869.s001.tif]
