# Supplementary material for: Effects of Early Life Paracetamol Use on the Incidence of Allergic Disease and Sensitization: 5 Year Follow-Up of an Ethiopian Birth Cohort
Source: PLoS One. 2014 Apr 9;9(4):e93869. doi: 10.1371/journal.pone.0093869 (PMC3981735; doi:10.1371/journal.pone.0093869)
Supplement: Table S1 — Summary of relevant variables collected at different time points in the cohort. (DOC) [file pone.0093869.s002.doc]

**Table S1**: Summary of relevant variables collected at different time points in the cohort

| **Variables** | **Follow up points** | | | | | |
| --- | --- | --- | --- | --- | --- | --- |
|  | **During pregnancy** | **At birth** | **At 2 months** | **Age 1** | **Age 3** | **Age 5** |
| ***Reported allergy outcomes*** |  |  |  |  |  |  |
| Wheeze symptom question |  |  |  | x | x | x |
| Eczema symptom question |  |  |  | x | x | x |
| Rhinitis symptom question |  |  |  |  | x | x |
| Reported/confirmed asthma |  |  |  | x | x | x |
| ***Socio-demographic characteristics*** |  |  |  |  |  |  |
| Age/sex/area of residence | x | x |  |  |  |  |
| Maternal occupation/education | x |  |  |  |  |  |
| Household income | x |  |  |  |  |  |
| ***Environmental risk factors*** |  |  |  |  |  |  |
| Roof/wall/floor type | x |  |  | x | x | x |
| Indoor smoking | x |  |  | x | x | x |
| Presence of animals | x |  |  | x | x | x |
| Insecticide use | x |  |  | x | x | x |
| Indoor/outdoor cooking | x |  |  | x | x | x |
| ***Family factors*** |  |  |  |  |  |  |
| Family history of allergy |  |  |  | x | x | x |
| Household size |  |  |  | x | x | x |
| Child’s place of sleep |  |  |  | x | x | x |
| Number of siblings |  |  |  | x | x | x |
| Birth order |  |  |  | x | x | x |
| ***Childhood characteristics*** |  |  |  |  |  |  |
| Breast feeding history |  | x | x | x | x |  |
| Vaccination |  | x | x | x | x |  |
| Use of paracetamol |  |  |  | x | x | x |
| Symptoms of respiratory tract infections (cough, fast breathing and fever) |  |  | x | x | x | x |
| ***Sensitization*** |  |  |  |  |  |  |
| Skin prick test (*D. pteronyssinus* and cockroach allergen) |  |  |  |  | x | x |
