# Supplementary material for: Effects of Early Life Paracetamol Use on the Incidence of Allergic Disease and Sensitization: 5 Year Follow-Up of an Ethiopian Birth Cohort
Source: PLoS One. 2014 Apr 9;9(4):e93869. doi: 10.1371/journal.pone.0093869 (PMC3981735; doi:10.1371/journal.pone.0093869)
Supplement: Table S2 — Distribution of potential confounders in the first year of life in relation to incident symptoms outcomes between ages 3 and 5. (DOC) [file pone.0093869.s003.doc]

**Table S2** Distribution of potential confounders in the first year of life in relation to incident symptoms outcomes between ages 3 and 5

| Variables | Wheeze free cohort  Overall  N (%) | Wheeze | Eczema free cohort  Overall  N (%) | Eczema | Rhinitis free cohort  Overall  N (%) | Rhinitis |
| --- | --- | --- | --- | --- | --- | --- |
|  |  | Crude OR  (95% CI) |  | Crude OR  (95% CI) |  | Crude OR  (95% CI) |
| Urban area of residence | 78 (11.5) | 0.39 (0.09,1.64) | 84 (12.0) | 0.38 (0.09,1.62) | 99 (12.4) | 0.48 (0.11,2.03) |
| Male gender | 336 (49.7) | 1.56 (0.81,2.99) | 362 (51.7) | 0.88 (0.46,1.68) | 406 (50.9) | 1.35 (0.65,2.80) |
| Maternal education (formal vs. non formal) | 121 (17.9) | 1.58 (0.75,3.32) | 133 (19.0) | 1.11 (0.50,2.47) | 146 (18.3) | 0.47 (0.14,1.56) |
| Low birth weight (<2.5kg) vs. normal | 28 (6.2) | 2.44 (0.68,8.81) | 37 (7.8) | 0.78 (0.18,3.41) | 39 (7.2) | 0.71 (0.09,5.48) |
| Cough reported at yr 1 | 390 (57.7) | 3.11 (1.40,6.87) | 436 (62.3) | 1.22 (0.62,2.43) | 485 (60.8) | 1.60 (0.73,3.54) |
| Fast breathing at yr 1 | 218 (32.3) | 2.46 (1.29,4.70) | 264 (37.7) | 1.16 (0.60,2.24) | 296 (37.1) | 1.42 (0.69,2.92) |
| Fever reported at yr 1 | 513 (75.9) | 6.44 (1.52,27.2) | 550 (78.6) | 1.53 (0.63,3.73) | 625 (78.3) | 4.16 (0.98,17.70) |
| Exclusive breast feeding at 2 mo | 565 (83.8) | 1.79 (0.62,5.13) | 592 (84.8) | 0.79 (0.34,1.84) | 670 (84.3) | 0.97 (0.36,2.57) |
| Vaccination at 2 mo | 393 (58.3) | 1.20 (0.62,2.33) | 415 (59.4) | 0.75 (0.39,1.44) | 470 (59.1) | 0.83 (0.40,1.72) |
| Parental allergic history | 33 (4.9) | 0.48 (0.06,3.64) | 37 (5.1) | - | 48 (6.0) | 1.71 (0.50,5.88) |
| Insecticide use in the home | 559 (82.7) | 1.20 (0.49,2.92) | 581 (83.0) | 2.55 (0.77,8.46) | 647 (83.3) | 0.54 (0.23,1.24) |
| Household size |  |  |  |  |  |  |
| 1-3 | 87 (12.9) | 1 | 81 (11.6) | 1 | 94 (12.1) | 1 |
| 4-6 | 370 (54.7) | 0.68 (0.30,1.58) | 403 (57.6) | 0.58 (0.22,1.52) | 435 (56.0) | 0.64 (0.20,2.03) |
| 7^+^ | 219 (32.4) | 0.37 (0.13,1.04) | 216 (30.9) | 0.93 (0.35,2.50) | 248 (31.9) | 1.35 (0.43,4.21) |
| No of older siblings |  |  |  |  |  |  |
| 0 | 96 (14.2) | 1 | 94 (13.4) | 1 | 105 (13.5) | 1 |
| 1-3 | 366 (54.1) | 1.19 (0.48,2.99) | 397 (56.7) | 1.38 (0.47,4.11) | 431 (55.5) | 1.06 (0.30,3.78) |
| 4-10 | 214 (31.7) | 0.51 (0.17,1.56) | 209 (29.9) | 1.37 (0.43,4.38) | 241 (31.0) | 2.10 (0.59,7.49) |
| Child’s sleeping place |  |  |  |  |  |  |
| Bed/platform | 52 (7.7) | 1 | 52 (2.4) | 1 | 61 (7.9) | 1 |
| Floor | 291 (43.1) | 1.40 (0.40,4.86) | 309 (44.2) | 2.78 (0.36,21.6) | 329 (42.4) | 1.71 (0.38,7.58) |
| Grass matting | 332 (49.2) | 0.72 (0.20,2.60) | 338 (48.4) | 3.38 (0.44,25.8) | 386 (49.7) | 0.78 (0.17,3.68) |
| Indoor cooking | 549 (81.2) | 1.10 (0.47,2.54) | 568 (81.1) | 1.62 (0.62,4.22) | 630 (81.1) | 0.93 (0.37,2.32) |
| Indoor kerosene use | 77 (11.4) | 1.40 (0.57,3.47) | 77 (11.0) | 0.66 (0.20,2.20) | 87 (11.2) | 1.62 (0.60,4.36) |
| Smoking in the house at yr 3 | 85 (12.6) | 0.35 (0.08,1.49) | 89 (12.7) | 0.56 (0.17,1.85) | 99 (12.4) | 1.05 (0.36,3.06) |
| Antibiotic use at yr 3 | 183 (27.1) | 1.02 (0.50,2.09) | 200 (28.6) | 1.80 (0.93,3.50) | 225 (28.2) | 1.04 (0.47,2.30) |
| Thatched roof vs. corrugated iron sheet | 528 (78.1) | 0.96 (0.45,2.07) | 542 (77.4) | 1.35 (0.59,3.13) | 609 (78.0) | 1.14 (0.46,2.82) |
