# Supplementary material for: Effects of Early Life Paracetamol Use on the Incidence of Allergic Disease and Sensitization: 5 Year Follow-Up of an Ethiopian Birth Cohort
Source: PLoS One. 2014 Apr 9;9(4):e93869. doi: 10.1371/journal.pone.0093869 (PMC3981735; doi:10.1371/journal.pone.0093869)
Supplement: Table S3 — Distribution of potential confounders in the first year of life in relation to incident sensitization between ages 3 and 5. (DOC) [file pone.0093869.s004.doc]

**Table S3** Distribution of potential confounders in the first year of life in relation to incident sensitization between ages 3 and 5

| Variables | Not sensitized up to age 3 (N=766) | | | |
| --- | --- | --- | --- | --- |
|  | Overall  N (%) | n (%) new sensitization | Crude OR  (95% CI) | p-value |
| Urban area of residence | 89 (11.6) | 1 (1.1) | 0.54 (0.07,4.15) | 0.55 |
| Male gender | 390 (50.9) | 8 (2.1) | 1.10 (0.40,3.08) | 0.85 |
| Maternal education (formal vs. informal) | 146 (19.1) | 2 (1.4) | 0.65 (0.14,2.91) | 0.57 |
| Cough reported at yr 1 | 474 (62.0) | 8 (1.7) | 0.70 (0.25,1.94) | 0.49 |
| Fast breathing at yr 1 | 283 (37.0) | 6 (2.1) | 1.14 (0.40,3.24) | 0.80 |
| Fever reported at yr 1 | 607 (79.4) | 9 (1.5) | 0.38 (0.13,1.09) | 0.06 |
| Exclusive breast feeding at 2 mo (vs. non exclusive BF) | 648 (84.9) | 11 (1.7) | 0.64 (0.18,2.35) | 0.50 |
| Vaccination at 2 mo | 448 (58.7) | 8 (1.8) | 0.94 (0.32,2.73) | 0.90 |
| Parental allergic history | 49 (6.4) | 1 (2.0) | 1.05 (0.13,8.13) | 0.97 |
| Insecticide use in the home | 617 (82.9) | 14 (2.3) | 2.93 (0.38,22.52) | 0.28 |
| Household size |  |  |  | 0.46 |
| 1-3 | 88 (11.8) | 1 (1.1) | 1 | 0.23† |
| 4-6 | 415 (55.8) | 7 (1.7) | 1.49 (0.18,12.32) |  |
| 7^+^ | 241 (32.4) | 7 (2.9) | 2.60 (0.31,21.59) |  |
| No of older siblings |  |  |  | 0.76 |
| 0 | 98 (13.2) | 2 (2.0) | 1 | 0.62† |
| 1-3 | 411 (55.2) | 7 (1.7) | 0.83 (0.17,4.07) |  |
| 4-10 | 235 (31.6) | 6 (2.8) | 1.26 (0.25,6.36) |  |
| Child’s sleeping place |  |  |  | 0.96 |
| Bed/platform | 57 (7.7) | 1 (1.8) | 1 |  |
| Floor | 315 (42.3) | 6 (1.9) | 1.09 (0.13,9.23) |  |
| Grass matting | 372 (50.0) | 8 (2.2) | 1.23 (0.15,10.05) |  |
| Indoor cooking | 607 (81.6) | 12 (2.0) | 0.90 (0.25,3.24) | 0.87 |
| Indoor kerosene use | 81 (10.9) | 1 (1.2) | 0.58 (0.08,4.47) | 0.60 |
| Smoking in the house at yr 3 | 102 (13.4) | 2 (2.0) | 1.00 (0.22,4.49) | 0.99 |
| Antibiotic use at yr 3 | 221 (28.9) | 4 (1.8) | 0.89 (0.28,2.83) | 0.85 |
| Thatched roof vs. corrugated iron sheet | 584 (78.0) | 12 (2.2) | 1.86 (0.41,8.32) | 0.41 |

† p value for trend
